# Optimizing Pretreatment Parameters for Enhanced Phosphorus Recovery from High-Phosphorus Wastewater via Nanofiltration

**DOI:** 10.3390/membranes15110331

**Published:** 2025-10-31

**Authors:** Guodong Wu, Lu Wang, Bing Qin, Fanbin Meng, Yonghu He, Xin Wang, Jing Bai, Jingpeng Zhang, Yuanhao Wang

**Affiliations:** 1 Center of New Energy Research, School of Intelligence Science and Technology (School of Future Technology), Xinjiang University, Urumqi 830047, China; 2 Department of Foreign Languages, Yantai Nanshan University, Yantai 264000, China; 3 SINOPEC Research Institute of Petroleum Processing Co., Ltd., Beijing 100080, China; 4 SHIHEZI JOINCHIN ELECTRODE FOIL Co., Ltd., Shihezi 832000, China; 5 Wanhua Chemical Group Co., Ltd., Yantai 600309, China; 6 School of Intelligence Science and Technology, Xinjiang University, Urumq 830046, China

**Keywords:** nanofiltration membrane, phosphorus recovery, pretreatment pH adjustment

## Abstract

The effects of pretreatment pH value, operating pressure, and concentration factors on the performance of nanofiltration membrane concentration and the recovery of phosphorus-containing wastewater were systematically studied. A novel pretreatment strategy using solid sodium hydroxide was developed to adjust the feed solution pH, achieving optimal solid removal and minimized conductivity at pH = 5. Unlike conventional calcium-based methods, this approach avoids excessive chemical sludge formation and mitigates membrane scaling, enhancing system stability. Experimental results demonstrate that both phosphorus rejection and desalination efficiency are significantly influenced by feed solution pH, operating pressure, and concentration ratio. While increasing pH and pressure improve total phosphorus (TP) rejection and desalination rates, these benefits are accompanied by reduced membrane flux due to elevated osmotic pressure and intensified concentration polarization. The membrane exhibited optimal performance at a feed pH of 5 and an operating pressure of 3 MPa, with sustained flux and enhanced separation efficiency. Under these conditions, when the wastewater was concentrated fivefold at 25 °C, the TP rejection rate and desalination efficiency reached 92.9% and 91.8%, respectively.

## 1. Introduction

Water eutrophication has become a significant global environmental issue, with phosphorus pollution identified as one of the primary contributors [1,2,3,4,5]. Driven by the rapid expansion of industrial capacity and urbanization in China, the discharge of phosphorus-containing wastewater has increased considerably, originating from industrial processes such as the production of phosphorus chemicals, electroplating, and fertilizers, as well as from domestic sewage due to the widespread use of phosphorus-based detergents [6,7,8]. However, excessive phosphorus discharge can cause algal blooms, which disrupt aquatic ecosystems, lead to biodiversity loss, and pose a threat to drinking water safety [9,10]. Traditional methods of phosphorus removal, such as biological treatment and chemical precipitation, are limited by instability and the potential for secondary pollution [11,12,13]. Consequently, the development of more efficient, cost-effective, and sustainable phosphorus removal technologies is urgently needed [14,15,16,17].

Membrane separation technologies, particularly nanofiltration [18,19,20], have attracted increasing attention due to their unique separation capabilities, which lie between those of reverse osmosis and ultrafiltration [21,22,23,24,25].

pH significantly affects treatment efficiency. Alkaline conditions enhance the ionization of phosphate ions, increasing the negative charge density on the membrane surface and strengthening electrostatic repulsion [26,27,28,29,30]. However, much of the existing research is limited to controlled laboratory settings, with limited exploration of complex, real-world wastewater systems [31,32,33,34]. Furthermore, issues such as membrane fouling and long-term operational stability remain largely unaddressed, hindering the wider adoption of nanofiltration technology.

The current research encounters several significant challenges [35,36]. Phosphorus removal efficiency remains insufficient, with existing membranes struggling to meet increasingly stringent discharge standards. Membrane fouling, caused by organic and colloidal substances in wastewater, rapidly reduces membrane flux. Kent et al. established a simplified pretreatment method integrating hydraulic mixing with brief flocculation (12–14 min) to replace the conventional coagulation–flocculation process, which consistently reduced total phosphorus from 5 mg/L to below 0.3 mg/L. This approach simultaneously reduces coagulant usage and significantly improves organic compound removal [37]. In addition, the optimization of process parameters, particularly the interactions between pH and other water quality factors, has not been systematically explored. These limitations result from an incomplete understanding of membrane–pollutant interactions and a lack of tailored process designs for specific wastewater characteristics. For example, in the treatment of phosphorus-laden wastewater, some studies adjust the pH to 8.5–10 by first adding calcium chloride and then using solid sodium hydroxide. It was observed that elevated calcium chloride dosage correlated with increased feed solution conductivity. However, the alkaline conditions facilitated the precipitation of divalent ions (e.g., Ca^2+^, Mg^2+^), resulting in accelerated membrane scaling and reduced operational longevity. Addressing these challenges requires both advances in separation mechanisms and the optimization of engineering processes [38,39,40].

Therefore, to further optimize the process parameters and improve the efficiency of the phosphorus recovery process, this work systematically explored the effects of pretreatment pH, operating pressure, and concentration factors on the concentration and recovery efficiency of nanofiltration membrane treatment of phosphorus-containing wastewater. The solid sodium hydroxide was used as a pH regulator to optimize the phosphorus recovery process by adjusting the acidity and alkalinity of the nanofiltration feed solution. The influence of different pH values on the removal rate and conductivity of water samples was investigated at first, and a quantitative relationship between pH value and membrane separation performance was obtained. Based on preliminary experimental results, the synergistic mechanism of pretreatment conditions, such as operating pressure and concentration ratio, on the nanofiltration concentration process was conducted, providing a scientific basis for the optimization and selection of process parameters.

## 2. Materials and Methods

### 2.1. Experimental Materials

Reagents: Sodium hydroxide, ammonium molybdate solution, and ascorbic acid, all of analytical purity.

Instrument: UV-9000s dual beam UV visible spectrophotometer (METASH, Shanghai Yuanxi Instrument Co., Ltd., Shanghai, China); magnetic stirrer (LICHEN, Hunan Lichen Instrument Technology Co., Ltd., Changsha, China). The nanofiltration membrane is a Dow NF90-1812 polyamide thin-film composite membrane (Guochu Technology (Xiamen) Co., Ltd., Xiamen, China) with an effective area of 1.2 m^2^. Key characterization parameters include the following: molecular weight cut-off (~200 Da), surface hydrophilicity (contact angle ~40°), and surface charge properties (isoelectric point at pH 3.6).

### 2.2. Analysis of Original Water Quality

The acidic wastewater (pH = 1.6) from SHIHEZI JOINCHIN ELECTRODE FOIL Co., Ltd. (Shihezi, Xinjiang Province, China) was investigated in this study, which was characterized by exceptionally high conductivity (17.3 mS/cm) and complex contaminant composition. Elevated concentrations of fluoride (2001 mg/L), phosphate (4577 mg/L as PO_4_^3−^), sulfate (4631 mg/L as SO_4_^2−^), and metal ions (82.3 mg/L Fe species, 573.0 mg/L Mg species) were contained in the wastewater, with total dissolved solids reaching 13 g/L. All target analytes were analyzed in accordance with standard methods: phosphorus content via the ammonium molybdate spectrophotometric method (GB 11893-89), fluoride ions via the ion-selective electrode method (GB 7484), and metal elements via inductively coupled plasma optical emission spectrometry (ICP-OES) pursuant to US EPA Method 6010D.

The strong acidic environment exceeds the tolerance range of conventional nanofiltration membranes, and high concentrations of miscellaneous salt ions can easily cause fouling of the nanofiltration membrane. Thus, pretreatment has become the key to the process, which requires measures such as pre-removal of impurity ions, reduction in conductivity, and regulation of the chemical environment of the solution to alleviate membrane fouling and flux attenuation caused by concentration polarization and scaling in the subsequent nanofiltration concentration process.

### 2.3. Experimental Device

**Figure 1:**
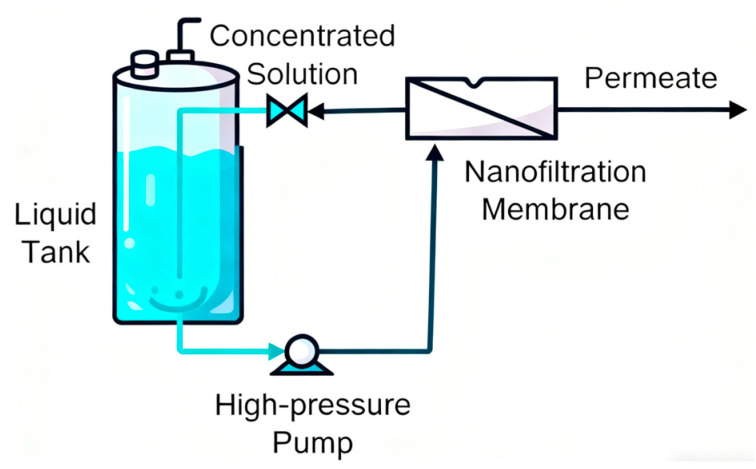
The nanofiltration membrane device.

The experimental system is schematically presented in Figure 1. The feed solution was initially introduced into the storage tank, from where it was transferred to the nanofiltration membrane module by means of a high-pressure pump. The feed flow rate was adjusted through precise regulation of the pump frequency, while the operating pressure of the nanofiltration unit (2–3 MPa) was controlled by systematic valve adjustment.

### 2.4. Experimental Method

The experimental procedure involved pretreating a small volume of feed solution by adjusting its pH with solid sodium hydroxide. The effects of varying pH levels on solid content removal and conductivity reduction were investigated.

Following pretreatment optimization, 6 L of feed solution were adjusted to the optimal pH, stirred at room temperature for 1 h, and filtered to remove impurities, ensuring solution clarity for nanofiltration. Nanofiltration experiments were conducted at pressures of 2 and 3 MPa, with the feed solution concentrated at different ratios. Samples of permeate and concentrate were collected at each concentration level for water quality analysis. Experimental conditions included a constant feed flow rate, an operating temperature of 25 °C, and concentration ratios ranging from 1 to 3 times.

The impact of pH, operating pressure, and concentration ratio on the nanofiltration process was systematically evaluated. Using optimized parameters, a 5-fold concentration experiment was performed on phosphorus-containing wastewater. Key performance indicators, including permeate flux, total phosphorus (TP) rejection rate, pH, and desalination rate, were measured to assess the efficiency and feasibility of the process.

### 2.5. Calculation Method

Phosphorus content was determined using the ammonium molybdate spectrophotometric method. The TP rejection rate (*R*) was calculated as follows:

*R* = 1 − *ρ*(TP)_1_/*ρ*(TP)_2_ (1)

where *ρ*(TP)_1_ and *ρ*(TP)_2_ represent the mass concentrations of TP in the permeate and feed solutions, respectively.

The desalination rate *D* was calculated using

*D* = 1 − *γ*_1_/*γ*_2_ (2)

where *γ*_1_ and *γ*_2_ are the electrical conductivities of the permeate and feed solutions, respectively.

Membrane flux *J* was determined by

*J* = *V*/(*At*) (3)

where *V* is the permeate volume, *A* is the effective area of the nanofiltration membrane, and *t* is the permeation time.

The solid removal rate *S* was calculated as

*S* = 1 − *ρ*_1_*/ρ*_2_ (4)

where *ρ*_1_ and *ρ*_2_ are the mass concentrations of solids in the pretreated liquid and the original feed solution, respectively.

The concentration ratio VRR was defined as

VRR = *V*_0_/*V*_R_ (5)

where *V*_0_ and *V*_R_ are the volumes of the feed solution and the concentrate, respectively.

The Henderson–Hasselbalch equation was employed to systematically predict and analyze the distribution characteristics of various phosphate species (H_3_PO_4_, H_2_PO_4_^−^, HPO_4_^2−^, PO_4_^3−^) in wastewater under different pH conditions.

For the H_3_PO_4_/H_2_PO_4_^−^ equilibrium

(6) $$\alpha_{H_{2}P{O_{4}}^{-}}=1/[1+10^{\left(pK_{a1}\text{-}\mathrm{pH}\right)}]$$

For the H_2_PO_4_^−^/HPO_4_^2−^ equilibrium

(7) $$\alpha_{{{\mathrm{HPO}}_{4}}^{2-}}=1/[1+10^{\left(pK_{a2}\text{-}\mathrm{pH}\right)}]$$

For the HPO_4_^2−^/PO_4_^3−^ equilibrium

(8) $$\alpha_{P{O_{4}}^{3-}}=1/[1+10^{\left(pK_{a3}\text{-}\mathrm{pH}\right)}]$$

where α is the distribution coefficient of the respective phosphate ion, $pK_{a1}$, $pK_{a2}$ and $pK_{a3}$ are the first, second, and third acid dissociation constants for phosphoric acid, and pH is the solution pH.

## 3. Results

### 3.1. Pretreatment

In this study, solid sodium hydroxide was introduced to regulate pH, while concomitant monitoring of solid content and conductivity revealed perturbations in ionic equilibrium. This equilibrium shift induced selective precipitation of dissolved species, resulting in changes in the properties of the water sample (Table 1). The results show that the solid removal rate reaches the peak value and the conductivity is minimized at pH = 5. Unlike traditional calcium-based pretreatment methods, the sodium hydroxide effectively removes impurities while avoiding the generation of excessive chemical sludge typically associated with calcium-based treatments. Moreover, this approach prevents membrane scaling caused by alkaline feed solutions, thereby extending the service life of the membrane. In summary, the pretreatment method not only enhances the efficiency of pretreatment but also reduces the risk of secondary environmental pollution.

### 3.2. The Influence of Pretreatment on Nanofiltration Concentration Process

**Figure 2:**
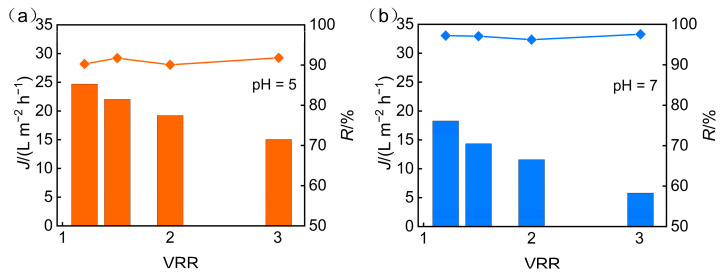
Impact of pretreatment pH on the following: (**a**) permeate flux; (**b**) TP rejection.

The pretreatment process was conducted by utilizing solid sodium hydroxide to adjust the pH values of the feed solution to 5 and 7, and the nanofiltration concentration experiments were carried out at a consistent operating pressure of 3 MPa. The impact of different pretreatment pH levels on the concentration effect of the nanofiltration membrane was assessed by comparing the experimental data like flux and TP rejection data obtained under varying pH conditions (Figure 2). The results demonstrate that nanofiltration membrane flux declines progressively as the pH of the feed solution rises, while TP rejection exhibits a marked improvement, primarily attributed to the effect of pH on the dissociation of phosphate ions.

**Figure 3:**
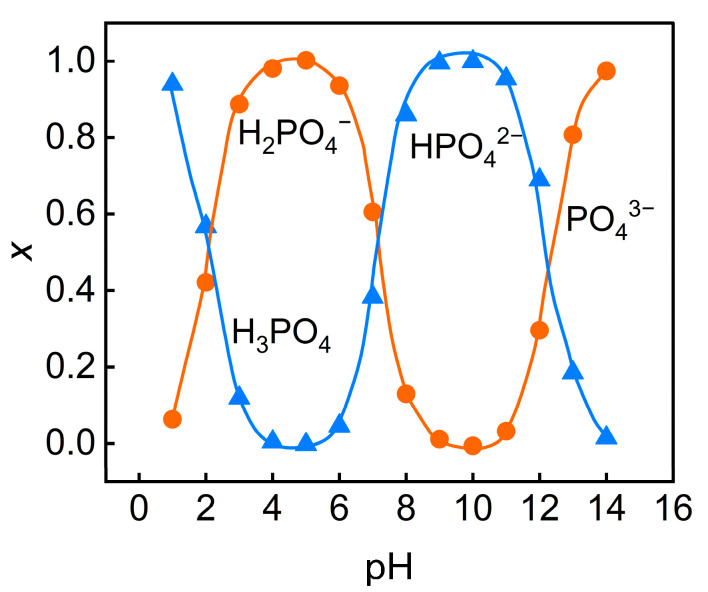
Relationship between pH variation and molar fractions of phosphoric ions.

In accordance with the Henderson–Hasselbalch equation, the degree of dissociation of phosphoric acid rises with the increasing pH. In the experiment, the initial pH of the feed solution was 1.6, at which time phosphorus predominantly existed in the form of H_3_PO_4_ molecules, while the concentrations of negatively charged H_2_PO_4_^−^, HPO_4_^2−^, and PO_4_^3−^ ions were extremely low. As the pH value increases, phosphate ions gradually dissociate into negatively charged ions. At pH = 5, phosphate ions mainly exist in the form of H_2_PO_4_^−^; when the pH value increased to 7, the concentrations of HPO_4_^2−^ and PO_4_^3−^ significantly increased (Figure 3). This dissociation behavior alters the form of phosphate ions in the solution, thereby influencing their migration and rejection on the surface of the nanofiltration membrane. The isoelectric point of the nanofiltration membrane (Dow NF90) used in this experiment is pH = 3.6. When the pH of the feed solution is higher than the isoelectric point, the membrane surface carries a negative charge and generates electrostatic repulsion with the dissociated phosphate anions, thereby increasing the rejection rate of TP. Although a higher pH enhances the TP rejection, it leads to adverse effects such as increased feed solution conductivity, higher osmotic pressure, and exacerbated concentration polarization. These issues result in diminished membrane flux, thereby hindering effective concentration recovery.

**Figure 4:**
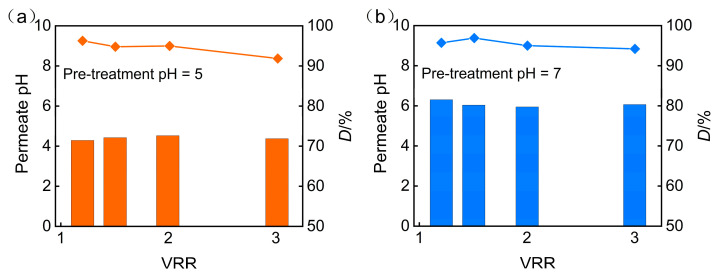
Impact of pretreatment pH on (**a**) permeate pH and (**b**) desalination efficiency.

Additionally, the increase in the pH of the feed solution will also impact the desalination rate of the nanofiltration permeate. Figure 4 illustrates the effect of pretreatment pH on the pH and desalination rate of nanofiltration permeate under an operating pressure of 3 MPa. It can be observed that the increase in the pH of the feed solution leads to an increase in the desalination rate. The increase in the pH of the feed solution enhances the surface charge density of the nanofiltration membrane and strengthens the electrostatic repulsion between the nanofiltration membrane and inorganic salt ions. Therefore, the desalination rate is higher at pH = 7. During the concentration process, the salt content in the feed solution increases, which enhances the shielding effect of inorganic salts on the surface charge of the nanofiltration membrane and weakens the electrostatic repulsion effect. Consequently, the desalination rate of the nanofiltration membrane slowly decreases with the increase in concentration ratio. On the other hand, an increase in the salt content of the feed solution causes swelling of the membrane pores, which may also reduce the desalination rate. Therefore, by adjusting the pH of the feed solution through pretreatment, the desalination rate increases, but as the concentration ratio increases, the desalination rate decreases.

In summary, adjusting the pH value of the feed solution in pretreatment has a significant impact on the concentration effect of the nanofiltration membrane. At pH = 5, the nanofiltration membrane achieved effective rejection of TP while maintaining a high flux. Although increasing the pH value to 7 can further improve TP rejection and desalination rates, the subsequent increase in osmotic pressure and decrease in flux limit its feasibility in practical applications. Therefore, considering the efficiency of concentration and recovery, a pH of 5 for the feed solution is more favorable for the treatment of phosphorus-containing wastewater by nanofiltration membranes.

### 3.3. The Influence of Pressure on the Concentration Process

**Figure 5:**
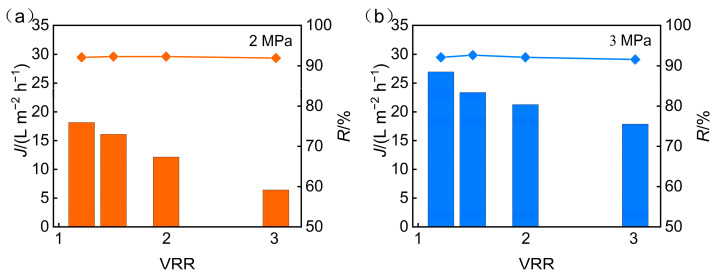
Operating pressure influence on the following: (**a**) permeate flux; (**b**) TP rejection.

Optimization of operating pressure is crucial for balancing high recovery, high concentration ratio, and low energy consumption. To refine process parameters and elucidate the regulatory role of pressure in membrane concentration, the influence of operating pressure on the performance of nanofiltration membranes was further explored. Figure 5 illustrates the impact of operating pressure on membrane permeate flux and TP rejection under conditions where the pH was maintained at 5 and the concentration ratios ranged from 1 to 3.

As the concentration ratio increased under constant pressure, both permeate flux and TP rejection gradually declined. This trend is likely due to the accumulation of solutes in the feed solution during concentration, which intensifies concentration polarization at the membrane surface. The resulting increase in mass transfer resistance contributed to the observed flux reduction.

An effective operational strategy to alleviate such flux decline involves the use of spacers as dedicated hydrodynamic elements to systematically optimize flow conditions near the membrane surface. Specifically, spacers mitigate solute-layer formation by creating localized turbulence that enhances interfacial mass transfer and thins the boundary layer [41]. This process helps counteract the increasing mass transfer resistance from solute accumulation and enables more stable flux over long-term operation. At the laboratory scale, spacer effects present a limited observable effect on system performance; however, in large-scale operations, design parameters such as spacer geometry and arrangement assume critical importance for determining process efficiency. Additionally, elevated solute concentrations weakened electrostatic repulsion at the membrane interface, thereby lowering the rejection rate of TP.

When the concentration factor remained unchanged, increasing the operating pressure led to higher flux and TP rejection rate. The enhanced flux is a result of increased driving force for water transport, while the improved TP rejection is likely due to stronger electrostatic interactions induced by elevated membrane surface charge under higher pressure. These interactions more effectively retained phosphorus within the retentate. However, when pressure increased from 2 MPa to 3 MPa, although the average flux rose by 65%, only a marginal gain in TP rejection was observed.

During the concentration process at constant pressure, increases in the concentration factor elevated the salinity of the feed solution. This further aggravated concentration polarization and weakened electrostatic interactions, leading to reduced TP rejection. In addition, salt accumulation on the membrane surface likely contributed to fouling, further impeding water flux and phosphorus separation performance.

**Figure 6:**
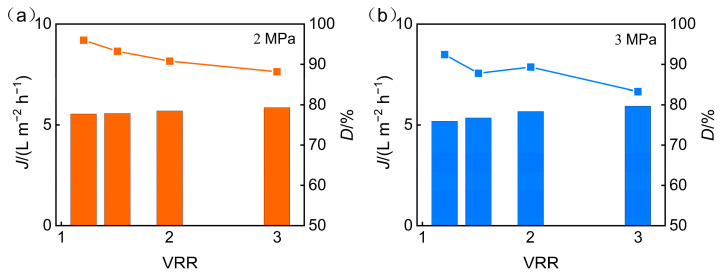
Operating pressure influence on the following: (**a**) permeate pH; (**b**) desalination efficiency.

Figure 6 illustrates the effect of operating pressure on the permeate pH and desalination performance of the nanofiltration membrane during the treatment of phosphorus-containing wastewater. With operating pressure increased, the permeate pH declined, while the desalination rate improved. When the concentration ratio remains constant, an increase in operating pressure enhances the membrane’s permeate flux, which boosts the convection, thereby not only improving the rejection rate of inorganic salts by the nanofiltration membrane but also leading to an increase in the desalination rate. Meanwhile, as the rejection of multivalent cations increases, additional H^+^ ions permeate the membrane to maintain electrochemical neutrality, leading to a slight decrease in permeate pH with rising pressure.

When the operating pressure is held constant, increasing the concentration ratio elevates the salinity of the feed solution. The higher ionic strength enhances the charge shielding effect, which in turn lowers the effective surface charge density of the membrane. As a result, electrostatic repulsion between the membrane and charged solutes such as phosphate ions is weakened, leading to reduced ion rejection and a decline in the membrane’s desalination efficiency.

To sum up, increasing operating pressure enhances TP rejection and desalination rates while enabling the nanofiltration membrane to sustain high flux over extended periods. Therefore, a feed solution pH of 5 and an operating pressure of 3 MPa are the optimal experimental parameters for the concentration and recovery of phosphorus-containing wastewater using the nanofiltration membrane in this study.

### 3.4. The Influence of Temperature Factor on the Concentration Process

**Figure 7:**
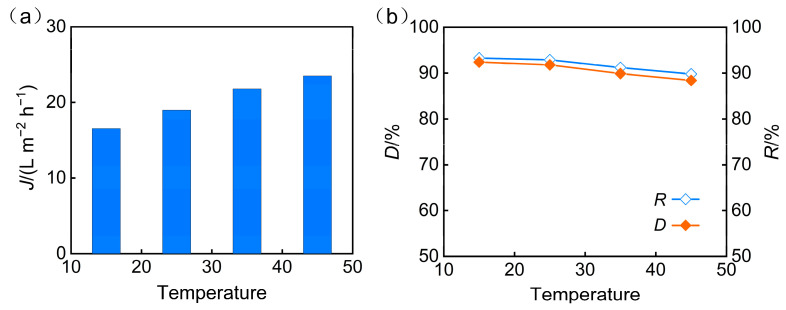
Operating temperature effect on (**a**) permeate flux and (**b**) desalination efficiency and TP rejection.

To investigate the effect of temperature on the nanofiltration performance for treating phosphorus-containing wastewater, experiments were conducted under fixed conditions of pH = 5, an operating pressure of 3 MPa, and a VRR of 3, across a temperature range of 15, 25, 35, and 45 °C. The results indicated that temperature increase had a dual effect on membrane separation performance. On one hand, the rise in water temperature reduced water viscosity, leading to a significant enhancement in membrane flux (Figure 7a). On the other hand, elevated temperature increased the mobility of polymer membrane segments and the diffusion capacity of solutes, potentially causing membrane pore swelling and weakening the sieving and electrostatic repulsion effects. For phosphate ions (primarily present as H_2_PO_4_^−^ at pH = 5) and other inorganic salt ions in this system, this weakening effect manifested as a gradual decreasing trend in TP rejection and desalination rates with increasing temperature (Figure 7b).

### 3.5. The Influence of Concentration Factor on the Concentration Process

**Figure 8:**
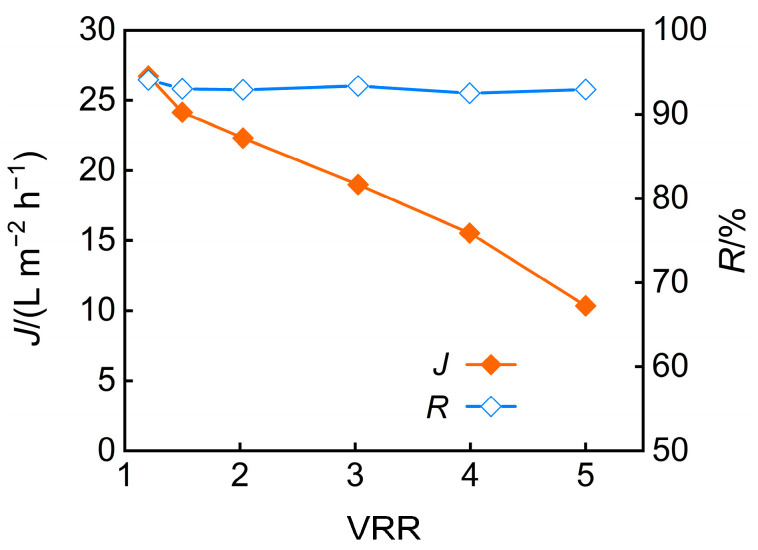
VRR effect on permeate flux and TP rejection.

The influence of the concentration factor on nanofiltration membrane performance was deeply studied. The effect of the concentration factor on permeate flux and TP rejection under conditions of pH 5 and an operating pressure of 3 MPa is illustrated in Figure 8. As the concentration factor increases, osmotic pressure rises, and membrane fouling intensifies, leading to a sustained reduction in permeate flux. The TP rejection rate remained almost unchanged, which can be attributed to two opposing factors. On one hand, the reduction in permeate flux weakened the convective effect of water, potentially leading to a decrease in TP rejection. On the other hand, the increase in fouling caused the membrane pores to narrow, thereby enhancing TP rejection.

**Figure 9:**
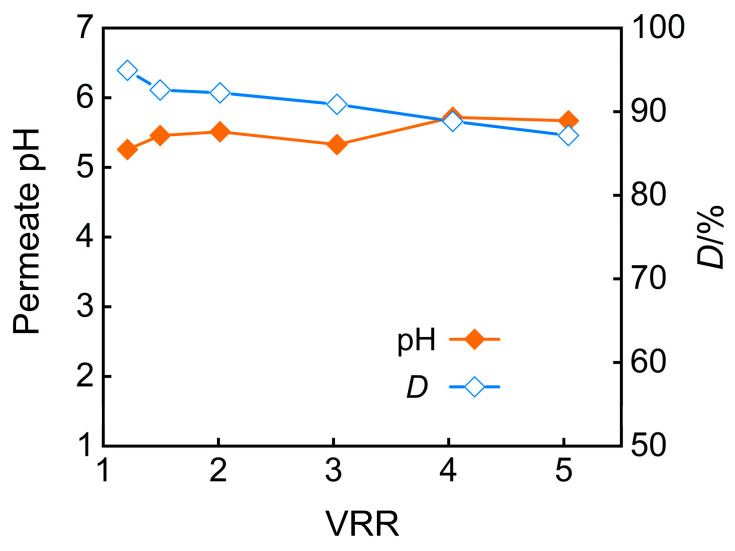
VRR effect on permeate pH and desalination efficiency.

The impact of the concentration factor on the pH and desalination rate of nanofiltration permeate is shown in Figure 9 when under conditions of pH 5 and an operating pressure of 3 MPa. The results reveal a declining desalination efficiency at higher concentration ratios, which are attributed to charge screening effects and membrane swelling from accumulated salts, coupled with an increase in permeate pH. Additionally, when the feed solution was concentrated fivefold, the TP rejection rate reached 92.9%, while the desalination rate achieved 91.8%.

**Figure 10:**
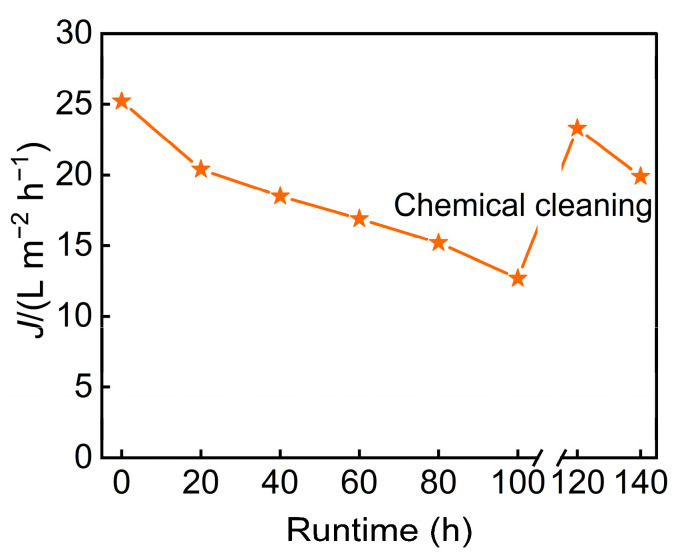
The relationship between runtime and membrane flux.

In addition, long-term continuous operation data (Figure 10) revealed a significant overall decline in membrane flux. The initial rapid attenuation was primarily attributable to intensified concentration polarization and membrane fouling. Following chemical cleaning, the flux can be almost restored to its initial level.

## 4. Conclusions

Systematic investigation established that nanofiltration membranes achieve optimal phosphorus recovery at a 3 MPa operating pressure with feed pH maintained at 5.0. Under these conditions, the system demonstrates stable operation with sustained water flux while maintaining high TP rejection and desalination efficiency. The pretreatment pH adjustment significantly influences separation performance through modulation of membrane surface charge characteristics and alteration of phosphate species distribution. While increased pH reduced membrane flux, both TP rejection and desalination rates are enhanced. In addition, increasing the operating pressure significantly enhances membrane flux and desalination efficiency. However, the effect on TP rejection is comparatively limited. When using a nanofiltration membrane to treat phosphorus-containing wastewater concentrated fivefold, the system maintains strong separation performance, achieving both high TP rejection and effective desalination. Under these optimized conditions, the TP rejection rate reaches 92.9% and the desalination rate achieves 91.8%. This work offers a general strategy that could guide the application of selective separation membranes in various resource recovery processes, including heavy metal recovery and concentration of high-value products.

## 5. Outlook

Although this study demonstrated the feasibility of enhancing TP recovery through feed solution pretreatment, it was limited by the absence of continuous-flow experiments and the constrained pH operating window. While the selected pH range achieved an optimal balance between membrane stability and anti-scaling performance, it restricted the exploration of separation efficiency under broader pH conditions. Therefore, future work will focus on developing continuous-flow experimental systems to evaluate long-term process stability, alongside pursuing the development of broadly pH-tolerant membrane materials to extend the applicability of nanofiltration technology in complex wastewater systems.

## Figures and Tables

**Table 1:** Effect of pretreatment pH on water samples.

pH	*ρ*_2_/(mg·L^−1^)	S/%	γ/(mS·cm^−1^)
1.6	12.8		18.3
5.0	4.5	64.8	7.5
7.0	5.3	50.0	21.8
8.0	7.5	40.2	24.9

## Data Availability

The original contributions presented in this study are included in the article. Further inquiries can be directed to the corresponding authors.

## References

[1] Powers S.M., Bruulsema T.W., Burt T.P., Chan N.I., Elser J.J., Haygarth P.M., Howden N.J.K., Jarvie H.P., Lyu Y., Peterson H.M. (2016). Long-term accumulation and transport of anthropogenic phosphorus in three river basins. Nat. Geosci..

[2] Yan X., Xia Y., Ti C., Shan J., Wu Y., Yan X. (2024). Thirty years of experience in water pollution control in Taihu Lake: A review. Sci. Total Environ..

[3] Miranda L.S., Wijesiri B., Ayoko G.A., Egodawatta P., Goonetilleke A. (2021). Water-sediment interactions and mobility of heavy metals in aquatic environments. Water Res..

[4] Hobbie S.E., Finlay J.C., Janke B.D., Nidzgorski D.A., Millet D.B., Baker L.A. (2017). Contrasting nitrogen and phosphorus budgets in urban watersheds and implications for managing urban water pollution. Proc. Natl. Acad. Sci. USA.

[5] Alori E.T., Glick B.R., Babalola O.O. (2017). Microbial Phosphorus Solubilization and Its Potential for Use in Sustainable Agriculture. Front. Microbiol..

[6] Tong Y., Zhang W., Wang X., Couture R.-M., Larssen T., Zhao Y., Li J., Liang H., Liu X., Bu X. (2017). Decline in Chinese lake phosphorus concentration accompanied by shift in sources since 2006. Nat. Geosci..

[7] Brownlie W.J., Sutton M.A., Cordell D., Reay D.S., Heal K.V., Withers P.J.A., Vanderbeck I., Spears B.M. (2023). Phosphorus price spikes: A wake-up call for phosphorus resilience. Front. Sustain. Food Syst..

[8] Jin X., Guo J., Hossain M.F., Lu J., Lu Q., Zhou Y., Zhou Y. (2024). Recent advances in the removal and recovery of phosphorus from aqueous solution by metal-based adsorbents: A review. Resour. Conserv. Recycl..

[9] Wang Y., Kuntke P., Saakes M., van der Weijden R.D., Buisman C.J.N., Lei Y. (2022). Electrochemically mediated precipitation of phosphate minerals for phosphorus removal and recovery: Progress and perspective. Water Res..

[10] Luo D., Wang L., Nan H., Cao Y., Wang H., Kumar T.V., Wang C. (2022). Phosphorus adsorption by functionalized biochar: A review. Environ. Chem. Lett..

[11] Wurtsbaugh W.A., Paerl H.W., Dodds W.K. (2019). Nutrients, eutrophication and harmful algal blooms along the freshwater to marine continuum. Wiley Interdiscip. Rev. Water.

[12] Bunce J.T., Ndam E., Ofiteru I.D., Moore A., Graham D.W. (2018). A Review of Phosphorus Removal Technologies and Their Applicability to Small-Scale Domestic Wastewater Treatment Systems. Front. Environ. Sci..

[13] Di Capua F., de Sario S., Ferraro A., Petrella A., Race M., Pirozzi F., Fratino U., Spasiano D. (2022). Phosphorous removal and recovery from urban wastewater: Current practices and new directions. Sci. Total Environ..

[14] Wilfert P., Kumar P.S., Korving L., Witkamp G.-J., van Loosdrecht M.C.M. (2015). The Relevance of Phosphorus and Iron Chemistry to the Recovery of Phosphorus from Wastewater: A Review. Environ. Sci. Technol..

[15] Kelly P.T., He Z. (2014). Nutrients removal and recovery in bioelectrochemical systems: A review. Bioresour. Technol..

[16] Zhang W., Li J., Sun H., Che W., Li J. (2021). A mixed-flow bioretention system amended with water treatment residuals to enhance nitrogen and phosphorus removal performance. Desalination Water Treat..

[17] Park T., Ampunan V., Maeng S., Chung E. (2017). Application of steel slag coated with sodium hydroxide to enhance precipitation-coagulation for phosphorus removal. Chemosphere.

[18] Zhao Y., Tong T., Wang X., Lin S., Reid E.M., Chen Y. (2021). Differentiating Solutes with Precise Nanofiltration for Next Generation Environmental Separations: A Review. Environ. Sci. Technol..

[19] Han S., Zhu J., Uliana A.A., Li D., Zhang Y., Zhang L., Wang Y., He T., Elimelech M. (2022). Microporous organic nanotube assisted design of high performance nanofiltration membranes. Nat. Commun..

[20] Mehta C.M., Khunjar W.O., Nguyen V., Tait S., Batstone D.J. (2014). Technologies to Recover Nutrients from Waste Streams: A Critical Review. Crit. Rev. Environ. Sci. Technol..

[21] Zhou Q., Sun H., Jia L., Wu W., Wang J. (2022). Simultaneous biological removal of nitrogen and phosphorus from secondary effluent of wastewater treatment plants by advanced treatment: A review. Chemosphere.

[22] Zhao W., Bi X., Peng Y., Bai M. (2022). Research advances of the phosphorus-accumulating organisms of Candidatus Accumulibacter, Dechloromonas and Tetrasphaera: Metabolic mechanisms, applications and influencing factors. Chemosphere.

[23] Peng L., Dai H., Wu Y., Peng Y., Lu X. (2018). A comprehensive review of phosphorus recovery from wastewater by crystallization processes. Chemosphere.

[24] Vu H.H.T., Khan M.D., Tran V.T., Quang D.V., Dao V.-D., Lee S., Ahn J.W., Jung S.-h. (2020). Use of Calcite Mud from Paper Factories in Phosphorus Treatment. Sustainability.

[25] Song K.-G., Cho J., Cho K.-W., Kim S.-D., Ahn K.-H. (2010). Characteristics of simultaneous nitrogen and phosphorus removal in a pilot-scale sequencing anoxic/anaerobic membrane bioreactor at various conditions. Desalination.

[26] Zhou M., Chen J., Yu S., Chen B., Chen C., Shen L., Li B., Lin H. (2023). The coupling of persulfate activation and membrane separation for the effective pollutant degradation and membrane fouling alleviation. Chem. Eng. J..

[27] Liu Y., Wang S., Huo J., Zhang X., Wen H., Zhang D., Zhao Y., Kang D., Guo W., Ngo H.H. (2024). Adsorption recovery of phosphorus in contaminated water by calcium modified biochar derived from spent coffee grounds. Sci. Total Environ..

[28] Rahman M.M., Salleh M.A.M., Rashid U., Ahsan A., Hossain M.M., Ra C.S. (2014). Production of slow release crystal fertilizer from wastewaters through struvite crystallization—A review. Arab. J. Chem..

[29] Qiu Q., Gao M., Zhou W., Xu Z., Kong C., Qiu L., Zhang S., Sun S. (2022). Enhanced Phosphorus Removal during Municipal Wastewater Treatment by the Biological Aeration Filter with Modified Steel Slags. J. Environ. Eng..

[30] Ji Y.-L., Gu B.-X., An Q.-F., Gao C.-J. (2017). Recent Advances in the Fabrication of Membranes Containing “Ion Pairs” for Nanofiltration Processes. Polymers.

[31] Deng L., Dhar B.R. (2023). Phosphorus recovery from wastewater via calcium phosphate precipitation: A critical review of methods, progress, and insights. Chemosphere.

[32] Koh K.Y., Zhang S., Chen J.P. (2020). Hydrothermally synthesized lanthanum carbonate nanorod for adsorption of phosphorus: Material synthesis and optimization, and demonstration of excellent performance. Chem. Eng. J..

[33] Penn C.J., Camberato J.J. (2019). A Critical Review on Soil Chemical Processes that Control How Soil pH Affects Phosphorus Availability to Plants. Agriculture.

[34] Du M., Zhang Y., Wang Z., Lv M., Tang A., Yu Y., Qu X., Chen Z., Wen Q., Li A. (2022). Insight into the synthesis and adsorption mechanism of adsorbents for efficient phosphate removal: Exploration from synthesis to modification. Chem. Eng. J..

[35] Marchetti P., Peeva L., Livingston A. (2017). The Selectivity Challenge in Organic Solvent Nanofiltration: Membrane and Process Solutions. Annu. Rev. Chem. Biomol. Eng..

[36] Ji Y., Qian W., Yu Y., An Q., Liu L., Zhou Y., Gao C. (2017). Recent developments in nanofiltration membranes based on nanomaterials. Chin. J. Chem. Eng..

[37] Citulski J., Farahbakhsh K., Kent F. (2009). Optimization of phosphorus removal in secondary effluent using immersed ultrafiltration membranes with in-line coagulant pretreatment—Implications for advanced water treatment and reuse. Can. J. Civ. Eng..

[38] Agboola O., Maree J., Kolesnikov A., Mbaya R., Sadiku R. (2014). Theoretical performance of nanofiltration membranes for wastewater treatment. Environ. Chem. Lett..

[39] Lee S., Kang T., Lee J.Y., Park J., Choi S.H., Yu J.-Y., Ok S., Park S.-H. (2021). Thin-Film Composite Nanofiltration Membranes for Non-Polar Solvents. Membranes.

[40] Nguyen Thi H.Y., Nguyen B.T.D., Kim J.F. (2020). Sustainable Fabrication of Organic Solvent Nanofiltration Membranes. Membranes.

[41] Liang Y.Y. (2025). Role of spacers in osmotic membrane desalination: Advances, challenges, practical and artificial intelligence-driven solutions. Process Saf. Environ. Prot..

